# Experimental Investigation on the Fire Resistance of Glued-In Rod Timber Joints with Heat Resistant Modified Epoxy Resin

**DOI:** 10.3390/ma13122731

**Published:** 2020-06-16

**Authors:** Liquan Luo, Benkai Shi, Weiqing Liu, Huifeng Yang, Zhibin Ling

**Affiliations:** 1 College of Civil Engineering, Nanjing Tech University, Nanjing 211816, China; llqroom9401@163.com; 2 School of Civil Engineering, Southeast University, Nanjing 211189, China; benkaishi@seu.edu.cn; 3 School of Civil Engineering, Suzhou University of Science and Technology, Suzhou 215011, China; zbling@mail.usts.edu.cn

**Keywords:** fire resistance, glued-in rod timber joint, pull-out test, heat resistant, modified epoxy resin

## Abstract

This paper presents an experimental evaluation of the fire resistance of glued-in rod timber joints using epoxy resin, with and without modification. A heat-resistant modified resin was designed by adding inorganic additives into the epoxy resin, aiming to improve the heat resistance. Joints that were made using the modified epoxy resin at room temperature showed a bearing capacity comparable to those with commercial epoxy resin. Twenty-one joint specimens with the modified epoxy resin and six with a commercial epoxy resin were tested in a fire furnace to evaluate the fire resistance. The main failure mode was the pull-out of the rod, which is typical in fire tests of this type of joints. As to the effects of the test parameters, this study considered the effects of adhesive types, sectional sizes, stress levels, and fireproof coatings. The test results showed that the fire resistance period of a joint can be evidently improved by modifying the resin and using the fireproof coating, as the improvements reached 73% and 35%, respectively, compared with the joint specimens with commercial epoxy resin. It was also found that, for all specimens, the fire resistance period decreased with an increase in the stress level and increased with an increase in the sectional sizes.

## 1. Introduction

In recent years, timber structures have experienced considerable development throughout the world, owing to their ‘green’ and low-carbon properties. Research on timber structures has become a significant focus worldwide, especially in North America and Europe, and even in China [1].

As is well-known, joints or connections are important aspects of timber structures [2,3]. Among the various types of joints, the glued-in rod joint shows excellent structural performances, with a pronounced bearing capacity and considerable slip stiffness, and it can be applied to high-rise and large-span timber structures [4,5,6]. It has been demonstrated that the glued-in rod/rebar joints showed superior feasibility and mechanical properties while they was applied to prefabricated beam-to-column connection [7,8].

Many experimental and theoretical studies on glued-in rebar timber joints have been carried out. Broughton and Hutchinson [9] investigated the influence of adhesive types on the behaviour of bonded steel rod joints and found that epoxy resin shows the best bearing capacity. Chans et al. [10] investigated the influence of geometric and material characteristics on the performance of glued-in rod joints. The joint performance showed evident differences when the joint was parallel [11] and perpendicular [12] to the grain directions. Azinović et al. [13] evaluated the pull-out strength of glued-in rods in cross-laminated timber and obtained the pull-out failure modes. Vallée and Adam [14] significantly shortened the curing time of glued-in rods in timber to minutes by using Curie particles. Ling et al. [15] proposed an empirical model for predicting the bond-slip behaviour of glued-in rebar joints, and also conducted pull-out tests by considering timber species, rod types, adhesive types, bonded length, glue-line thickness, and angle with respect to the grain [16]. Fragiacomo and Batchelar [17,18] presented a design method for evaluating joint strength under the combined action of moment and axial forces, and also evaluated the long-term performance of glued-in steel rods. Xu et al. [19] researched the effects of manufacturing defects on the pull-out behaviour of glued-in rods, and concluded that the positioning defects of the rods can be negligible. Grunwald et al. [20,21] investigated the glued-in rod joints which were applied to engineered hardwood products, and found that the shear strength and transverse (tensile) strength of wood significantly influence the bearing capacities of joints, while longitudinal tensile strength proved to be less significant. Similarly, He & Xiao [22] also demonstrated that the glued-in rod joints can be applied to glued laminated bamboo structures, and showed significant mechanical properties in bearing capacity and slip stiffness.

Hence, research on glued-in rod joints at room temperature is comprehensive and detailed. However, considering the importance of the structural behaviour of timber joints under fire conditions, it is extremely urgent to investigate their fire resistance [23]. Some studies have researched the fire resistance of some traditional joints, such as tooth plate joints [24], bolted joints [25,26,27,28,29], and dowel joints [28,29,30,31]. Liu et al. [32] investigated the bonding performance of glulam with different adhesives at elevated temperature, and found bonding performances of structural adhesives deteriorated linearly with increasing temperature.

To improve the fireproof ratings of timber members, surface impregnation treatment [33] and encapsulation coating system are common methods, which were suggested in available design methods [34,35]. As to the surface impregnation treatment, two-hour fireproof performance can be obtained for cross laminated timber while it was covered with fire-retardant impregnated wood [36]. In addition, Yue et al. [37] improved mechanical properties and combustion performance of Chinese fir by using impregnation and densification methods. The encapsulation coating system includes the fire-retardant coating, gypsum boards, and suspended membrane-type encapsulation [38]. Popescu and Pfriem [39] summarised the existing investigations about treatments and modification to improve the fire resistant capacity of wood and wood based products. Applying concrete slabs to the top of timber joists was an effective method to improve the fireproof performance of timber members [40]. In addition, for the fire design of timber structures, the reduction section method by considering the char rating of timber was adopted in available standards [41,42].

However, only a few research studies have focused on the fire resistance of glued-in rod/rebar joints. Lartigau et al. [43] studied the influence of temperature on the mechanical properties of glued-in rebar timber joints with epoxy resins. The study found that the critical temperature was 58 °C, and the pull-out strength evidently decreased when the temperature surpassed 58 °C. Harris [44] performed pull-out experiments for glued-in rebar joints in laminated veneer lumber (LVL) by considering a hybrid adhesive composed of urethane methacrylate and cement, aiming to improve the fire resistance of epoxy resin. However, the structural performance of the proposed modified adhesives in room conditions was not very satisfactory. Park et al. [45] carried out fire experiments on glued-in rebar joints in which two epoxy resins and a composite adhesive (HY150) were adopted, and the experimental results showed that the residual strength of epoxy at 100 °C was reduced by 30% from its cold strength, whereas the composite adhesive showed a smaller strength loss. In conclusion, the fire-resistant adhesive of glued-in rod joints proposed in the existing researches showed the obvious disadvantage in bearing capacity at room temperature.

According to the available literatures, the existing researches on the fire-resistance of glued-in rod joints has mainly focused on those with a traditional and commercial epoxy resin. Only a few heat resistant modified adhesives have been proposed [44,45]. For this reason, this study proposed a type of heat resistant modified waterborne epoxy resin by introduction of an inorganic refractory filler aiming to improve the fire resistant performance of adhesive used in glued-in rod joints. Firstly, preliminary pull-out tests of the modified epoxy resin were performed at room temperature, and the test results showed satisfactory mechanical behaviours compared with the commercial epoxy resin. Subsequently, fire tests were conducted under insurance service office- (ISO-) fire conditions. Testing results showed that the glued-in rod joints using heat resistant modified epoxy resin showed evident improvement in fire-resistant time in fire tests, without significant decline in mechanical performance at room temperature.

## 2. Materials

### 2.1. Timber 

The wood species used in this work is Douglas fir glued-laminated (glulam) timber. Its mechanical properties are listed in Table 1. The compressive strength and elastic modulus were tested according to ASTM D198 [46]. The tensile strength was tested as 28.6 MPa according to EN 408 [47]. The adhesive applied to glulam was phenol resorcinol formaldehyde (PRF), which was a type of weathering adhesive that can be used in outdoor components and buildings [48]. The shear strength of glulam with adhesive bond line was tested as 9.2 MPa according to ASTM D905 [49]. The average moisture content and average density of the specimens were tested as 12.4% and 483 kg/m^3^, respectively, in accordance with EN 13183-1 [50].

### 2.2. Threaded Rods

The threaded rods adopted in this study were grade 8.8, which were manufactured in accordance with GB/T 15389 [51]. The thread pitch was 2 mm. The material properties of the threaded rods were obtained by tensile tests according to GB/T 228 [52]. Table 2 presents the material properties of the tested threaded rods.

### 2.3. Adhesives

The adopted adhesives included the heat resistant modified epoxy adhesive proposed by the authors and a type of commercial epoxy resin. The material properties of the commercial epoxy resin, as provided by the manufacturer MKT123 (Nanjing, China) brand, are shown in Table 3.

The modified epoxy adhesive is an innovative epoxy resin created by adding an additive. The additive mainly includes an inorganic adhesive and a refractory filler. Therefore, the modified epoxy adhesive used in this study includes two components: an epoxy resin base material and the inorganic adhesive. The material ratios of each component are listed in Table 4. The ‘portion’ in Table 4 denoting that one modified epoxy can be made of 100 g epoxy E51, 50 g water-borne epoxy resin, 10 g Dibutyl phthalate (DBP) flexibilizer, and so on. The modified epoxy adhesive showed good fluidity and price advantages in terms of application, as all the ingredients were cheap and easily available in the market.

### 2.4. Fire-Retardant Coating

To evaluate the improvement of modified epoxy adhesive proposed by the authors in fire resistant performance and the synergistic effect between modified epoxy adhesive and fire-retardant coatings, two types of fire-retardant coatings were employed to the some specimens in fire tests. The adopted fire-retardant coatings include ZN-1 fire-retardant paint and ZOAN200 waterborne fireproof paint.

ZN-1 fire-retardant paint was a type of white coating, consisting of inorganic flame-retardant agent, foaming agent, carbonizing agent, synthetic emulsion, titanium dioxide, additives, etc. According to GB 12441 [53], its fire-resistant time exceeds 20 min.

ZOAN200 waterborne fireproof paint was a type of colourless coating, which can be applied to the surface of the timber to provide fire protection and retain the grain of the wood. Its flame-retardant performance conforms to grade B1 (difficult-flammability) in accordance with GB 12441 [53]. Its main components include film forming emulsion, phosphorous-nitrogen-carbon flame-retardant materials, and titanium dioxide.

## 3. Glued-in Rod Joint Specimens

### 3.1. Geometry

**Figure 1:**
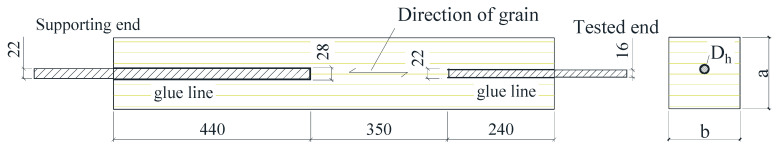
Geometry of the specimen (Unit: mm).

Figure 1 shows the configuration of a pull-pull specimen of the glued-in rod timber joint that was used for fire testing. The specimens were designed with double ends for testing. The tested end adopted an anchorage length of 15 d (d = the diameter) and a 3 mm thickness for the glue line. The diameter of the threaded rod at the tested end was 16 mm, whereas that at the supporting end was 22 mm. As shown in Figure 1, *D*_h_ was equal to 22 mm and represented the hole diameter at the centre of the timber block for the tested end. For all specimens, the supporting ends were designed with a greater capacity when compared with the tested end. For the supporting end, the anchorage length was equal to 20 d, and it was also protected by bulk ceramic fibre in the fire furnace, to ensure the anchorage reliability of the supporting end before the shear-bond failure of the tested end in the fire testing process.

### 3.2. Fabrication of Specimens

As shown in Figure 1, holes for the tested end and supporting end have a 22 mm and 28 mm diameter, respectively. The holes were manually drilled parallel to the grain, at both ends of the timber blocks. Each threaded rod was cleaned with acetone to remove dirt and grease from its surface before gluing. Structural adhesive was injected into the pre-drilled holes from the bottom, using a dosage of approximately 1/2 the volume of the hole. The cleaned threaded rods were then slowly inserted into the partially-filled holes and were rotated until they reached the bottom of the holes, to thereby expel any trapped air bubbles that might affect the bonding strength. To ensure the threaded rod implantation remained centered, a plastic casing with a 22 mm outside diameter, 3 mm wall thickness, and 10 mm length was employed on the top of the rod after insertion for the tested end. Similarly, the plastic casing with a 28 mm outside diameter were applied on rod at the supporting end. All specimens were kept in a room at 20 °C and at a relative humidity of 65% for at least 30 days before testing.

## 4. Bonding Behaviour at Room Temperature

**Figure 2:**
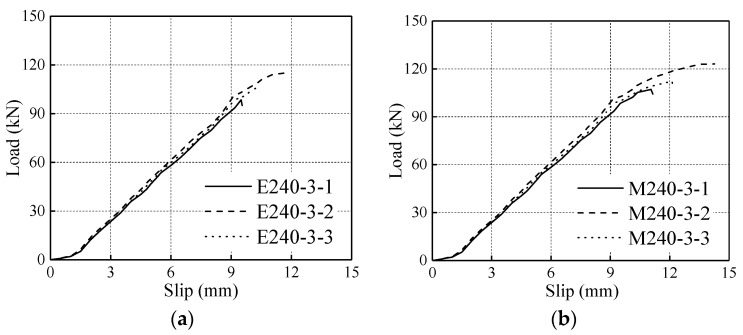
Load-slip curves at room temperature. (**a**) Specimens with commercial epoxy resin; (**b**) specimens with modified epoxy resin.

To investigate the bonding strength of the modified epoxy adhesive, pull-out tests were conducted at room temperature. The modified epoxy adhesive and commercial epoxy resin were adopted separately by the pull-pull specimens. Three samples were prepared for each type of adhesive. For all specimens, the anchorage length was 240 mm, whereas the thickness of the glue line was 3 mm. The anchorage length and glue line thickness were consistent with that of the specimens in the fire testing. The results of the pull-out tests at room temperature are depicted in Figure 2. Of particular note is that the values of slip tested in pull-out tests at room temperature were the displacement of the actuator aiming to being consistent with the measurement scheme in fire tests, instead of the interface relative slip between rod and timber which were commonly adopted in available pull-out tests at room conditions [15].

The average bearing capacity for the specimens with traditional commercial epoxy resin (E240-3) is 106.8 kN, whereas that for specimens with the modified epoxy adhesive (M240-3) is 114.2 kN. In addition, two groups of slip curves showed similar slopes denoting that the slip stiffness for the glued-in rod joint specimens with the two types of adhesives adopted has no obvious difference. 

**Figure 3:**
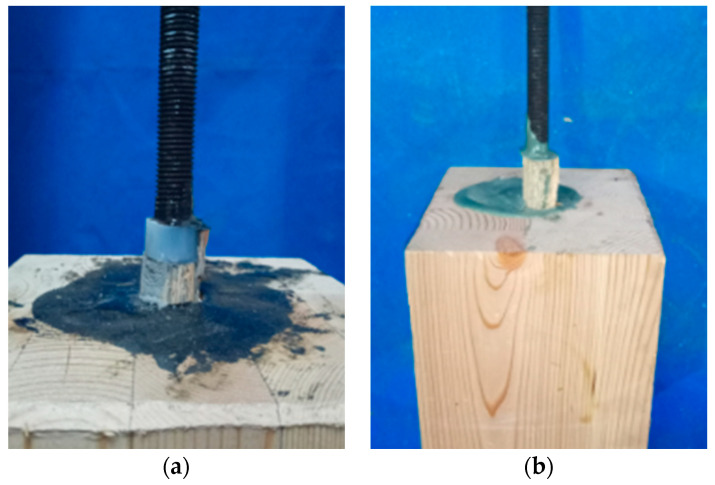
Failure modes of pull-out tests at rood temperatures. (**a**) Specimens with modified epoxy adhesive; (**b**) Specimens with commercial epoxy resin.

Figure 3 shows the failure photographs of tested glued-in rod joints. The specimens with heat resistant modified epoxy adhesive and commercial epoxy resin showed a same failure mode that was the shear failure of timber. In addition, the commercial epoxy resin showed the dark green as shown in Figure 3b, whereas the modified epoxy adhesive was almost black as shown in Figure 3a as the result of the application of cement and carbon fibre powders (see Table 4). Therefore, the modified epoxy adhesive shows similar mechanical performances compared with commercial epoxy resin at room temperature. Thus, the modified epoxy adhesive used in this study can be applied to glued-in rebar/rod timber joints in practical engineering in terms of mechanical behaviour at room temperatures.

## 5. Grouping and Test Facility for Fire Tests

### 5.1. Specimen Grouping

As shown in Table 5, the specimens were divided into nine groups. The diameter and anchorage length of the rod in all groups of specimens were 16 mm and 240 mm, respectively. Three specimens were prepared for each grouping of glued-in rod joints. The specimen designations were defined as Z/S/SF/ZF/ ZTF + section dimensions-stress level. Here, Z represents the specimens with the modified epoxy adhesive, S represents the specimens using the commercial epoxy resin adhesive, F represents the specimens with white fireproof coating, and TF represents the specimens with colourless fireproof coating. The stress level is the specific value of the applied load in the fire tests. The stress levels considered included 20%, 30%, and 50% of the ultimate bearing capacity, which were tested in room temperature conditions, as illustrated in Figure 2. For the Z series specimens with 20%, 30%, and 50% stress levels, the applied loads during fire tests were 22.8 kN, 34.3 kN, and 57.1 kN, respectively. For the specimens in groups S160 and SF160, the applied constant load during fire tests was 32.1 kN.

**Figure 4:**
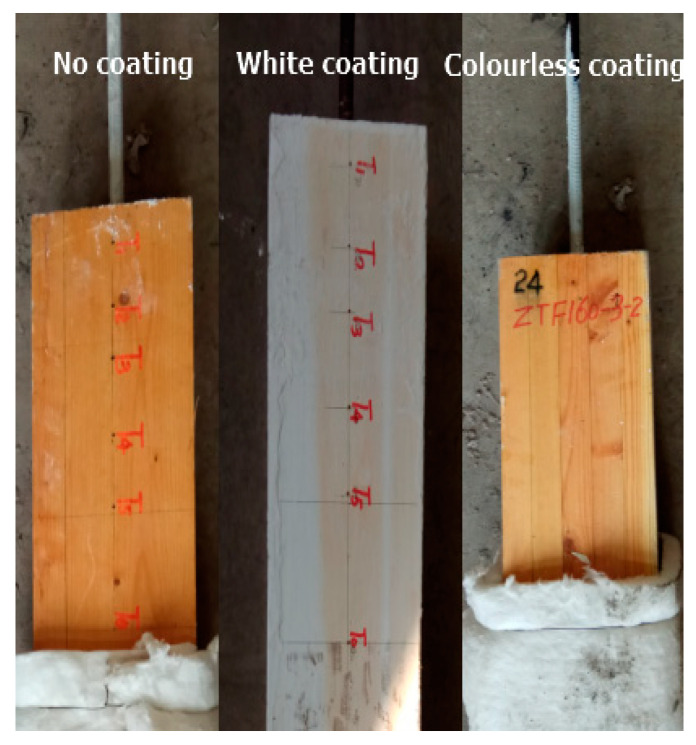
Specimens with different coating conditions.

For each group of specimens, three duplications were constructed and employed in the fire experiments. The influences of coatings and section dimensions were also investigated. Two types of fire-retardant coatings were employed, including ZN-1 (white coating) and ZOAN200 (colourless coating). Figure 4 depicts the glued-in rod timber joint specimens with a white coating, with a colourless coating, and without the coating.

### 5.2. Test Instrumentation

**Figure 5:**
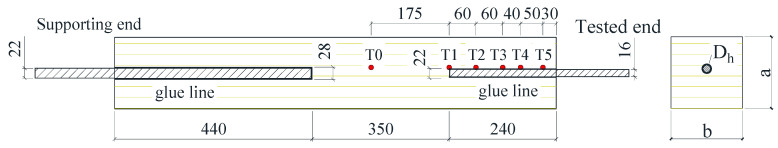
Temperature measurement position (Unit: mm).

Figure 5 illustrates the design and configuration of the temperature measurement positions. To better monitor the temperature variations inside the joint during the process of the fire tests, six temperature measurement points were installed in each specimen. The temperatures at corresponding positions along the rod were measured by thermocouples, denoted as T1, T2, T3, T4, and T5 in Figure 5. The temperature of the timber at the centre position was measured by thermocouple T0. 

In particular, holes of 3 mm in diameter were pre-drilled in designed positions of thermocouples for each specimen, and then the thermocouples were reliably banded into the target depth of the holes. From Figure 4, the 3 mm holes for thermocouples can be observed at the side face of glue-in rod joint specimens. The holes were sealed tightly with refractory clay after all thermocouples were fixed.

### 5.3. Testing Facilities 

**Figure 6:**
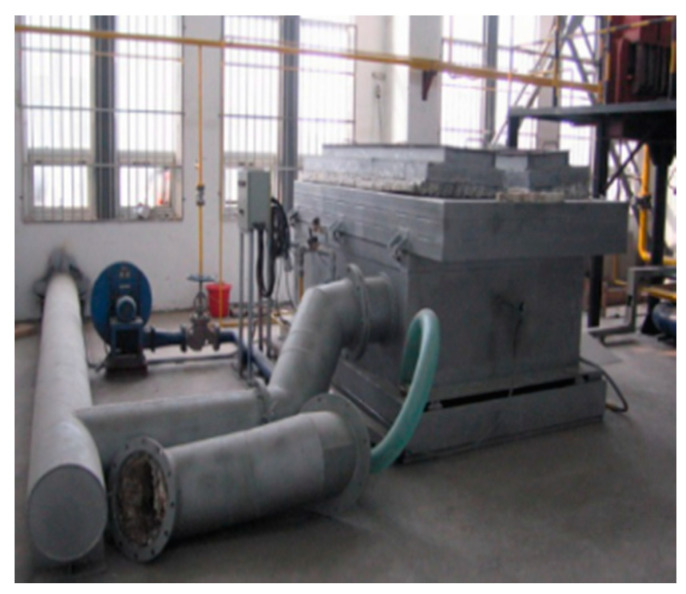
Fire furnace.

Fire tests for the glued-in rod timber joints were carried out in a small multipurpose fire-test furnace, as shown in Figure 6. The chamber size of the furnace was 1.8 m × 1.2 m × 0.5 m, and two thermocouples were employed to obtain the temperature inside the furnace. The practical furnace temperatures followed the ISO 834-1 [54] standard curve in the tests. The measurement frequency of the collection devices was two times per minute.

**Figure 7:**
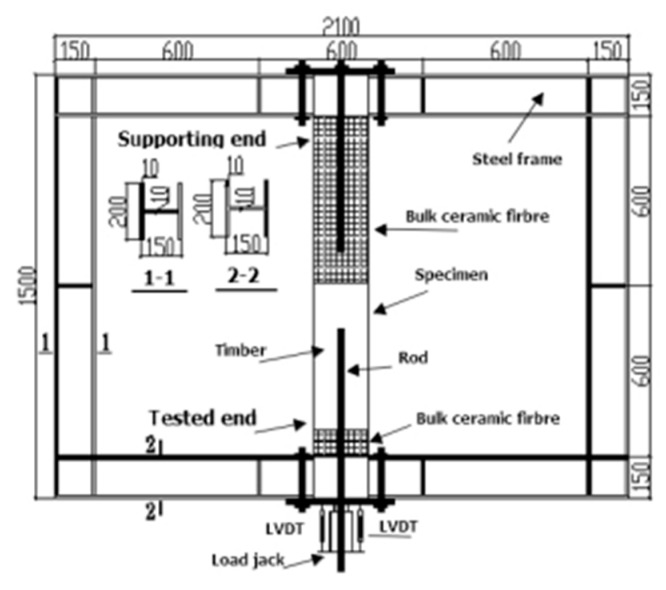
Loading frame (Unit: mm).

According to the furnace size and specimen installation requirements, a loading steel frame was used to apply a constant load during the fire experiments. The supporting end of each specimen was anchored on the steel frame by bolts, and the tested end was connected to the load jack. The designed shape and dimensions of the loading steel frame are illustrated in Figure 7.

To measure the relative slip between the rod and timber when a specimen was exposed to fire, a guyed displacement meter was adopted in the tests, and was firmly fixed on the tested rod. The specimen was deemed a failure when a target value for a certain stress level could not be maintained, or when an abrupt excess displacement occurred. The charring rate was calculated on the basis of experimental data from fire tests. The procedure for conducting the fire-resistance test for the glued-in rod timber joint was as follows:

- Clamp the specimen in the furnace, and cover the supporting end with fireproof cotton.
- Install the thermocouples, the loading system, and the guyed displacement meters.
- Apply the load at the designed stress level for more than 10 min.
- Ignite, and then maintain the load level until failure.
- Shut down the burners quickly after failure.
- Halt the wood charring by spraying water onto the specimens.

## 6. Experimental Results

### 6.1. Summary of Experimental Results

The failure time of fire tests for glued-in rod joints are presented in Table 6. During the fire test, the applied load was maintained by the oil pressure pump. When the interfacial temperature between the rod and adhesive rises to the critical value, the specimen slips greatly. At the meantime, the oil pressure pump cannot maintain a constant load, and the applied load decline abruptly. Thus, the specimen was considered to be damaged, and the failure time of individual specimen was determined accordingly.

It can be seen from Table 6 that all of the parameters, including the adhesive types, sectional sizes, stress levels, and fireproof coatings, showed at least one certain effect on the fire resistance period of the joints. From Table 6, the detailed results are summarized as follows:

- At the stress level of 30%, when the modified epoxy resin was used for group Z160-3, the average failure time reached 64 min. As a comparison, the average failure time was 37 min for group S160-3 with a traditional epoxy resin, indicating an improvement of 73.0%.
- Comparing group Z160-2 with group Z160-5, the failure time declined from 70 min to 57 min, whereas the stress level increased from 20% to 50%.
- At the stress level of 30%, the introduction of the white coating showed improvements of the fire resistance period by 54.1% and 20.3% for the traditional and modified epoxy resins, respectively.
- As the joint edge distance changed from 5 d (see Group Z160-3) to 7.5 d (see Group Z240-3), the failure time increased from 64 min to 83 min, i.e., an improvement of 29.7%.

Notably, for the specimens Z200-3-3 and SF160-3-3, an unanticipated breakage of the rod occurred. Therefore, they were not considered when calculating the average values of the fire resistance period within those groups.

### 6.2. Test Phenomena and Failure Modes

The glued-in rod joint specimens mainly failed as a pull-out failure of the rod, owing to the high temperature at the rod/adhesive interface. During the first 20 min, the relative slip and inner temperature rose slowly. Then, the temperature increased moderately over time, with slight fluctuations. The timber members began to burn with the evaporation of the moisture in the specimens. Once the interfacial temperature reached a certain value, a large slip immediately appeared.

**Figure 8:**
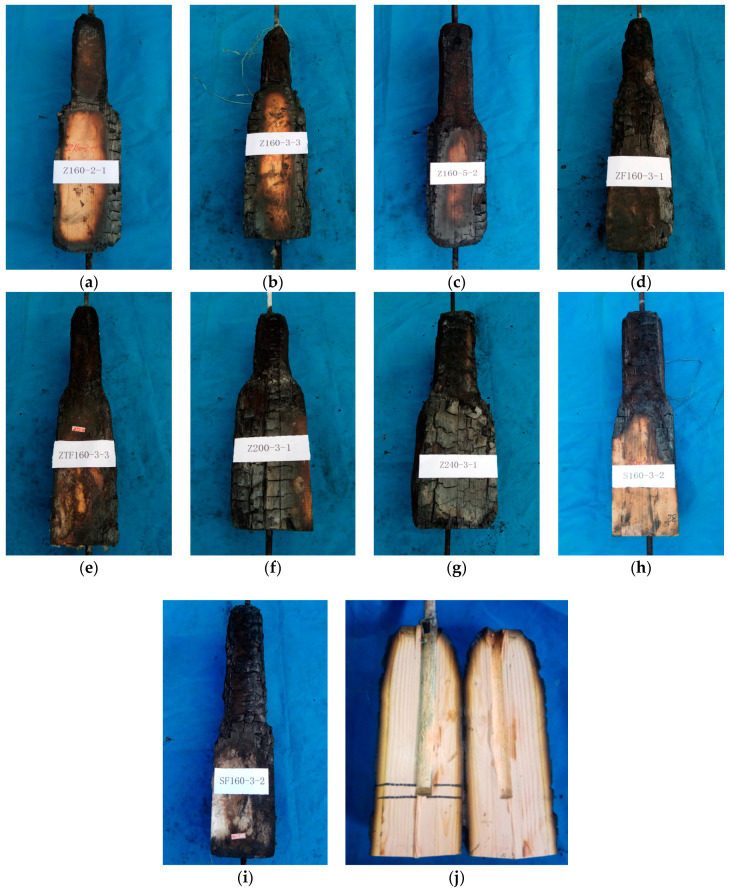
Failure modes of tested specimens. Surface charring for group (**a**) Z160-2; (**b**) Z160-3; (**c**) Z160-5; (**d**) ZF160-3; (**e**) ZTF160-3; (**f**) Z200-3; (**g**)Z240-3; (**h**) S160-3; (**i**) SF160-3; and (**j**) relative slip between rod and timber.

As shown in Figure 8a–i, the cross-section of the timber members was evidently decreased. The reduction of the cross-section depended on the char rating and fire time. For the specimens with fire-retardant coatings, the loss of cross section was relatively small. Group S160-3 specimens also showed the relatively complete cross section due to the shortest fire time. All specimens except Z200-3-3 and SF160-3-2 showed the evident slip, which can be observed in Figure 8j.

### 6.3. Slip-Time Curves

**Figure 9:**
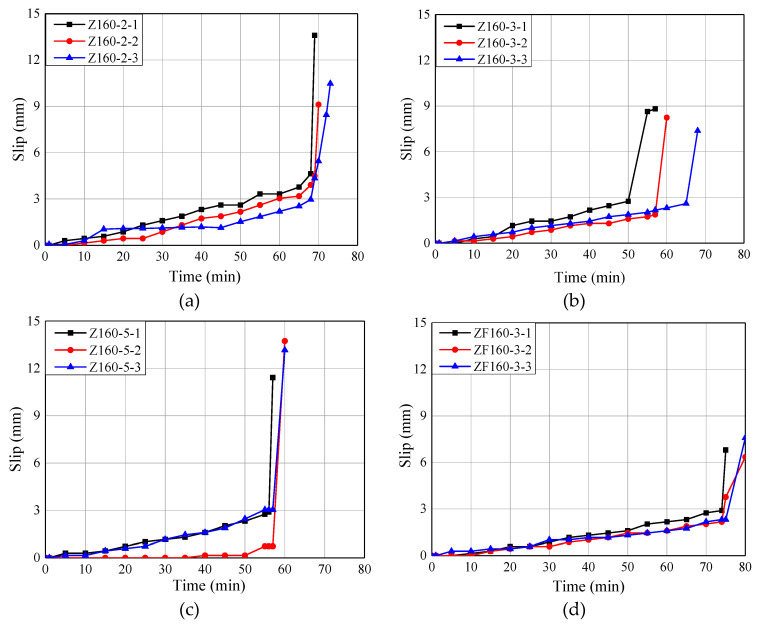
Slip-time curves. (**a**) Stress level = 20%. (**b**) Stress level = 30%. (**c**) Stress level = 50%. (**d**) Stress level = 30% with fireproof coating.

The relative slips at the loading end were recorded, and the slip-time curves are presented in Figure 9. In Figure 9, it can be seen that the relative slip increased gradually over time during the initial stage of the test. The maximum slip value ranged from 2.4 mm to 3.1 mm just before the final failure. At the final stage, the slip increased sharply, indicating the failure of the bonding interface. In addition, the fire resistance time of glued-in rod joints declined as the increase of stress levels. However, the fireproof coating can pronouncedly improve the fire resistance time of the glued-in rod timber joints by approximately 20%.

### 6.4. Temperature-Time Curves

**Figure 10:**
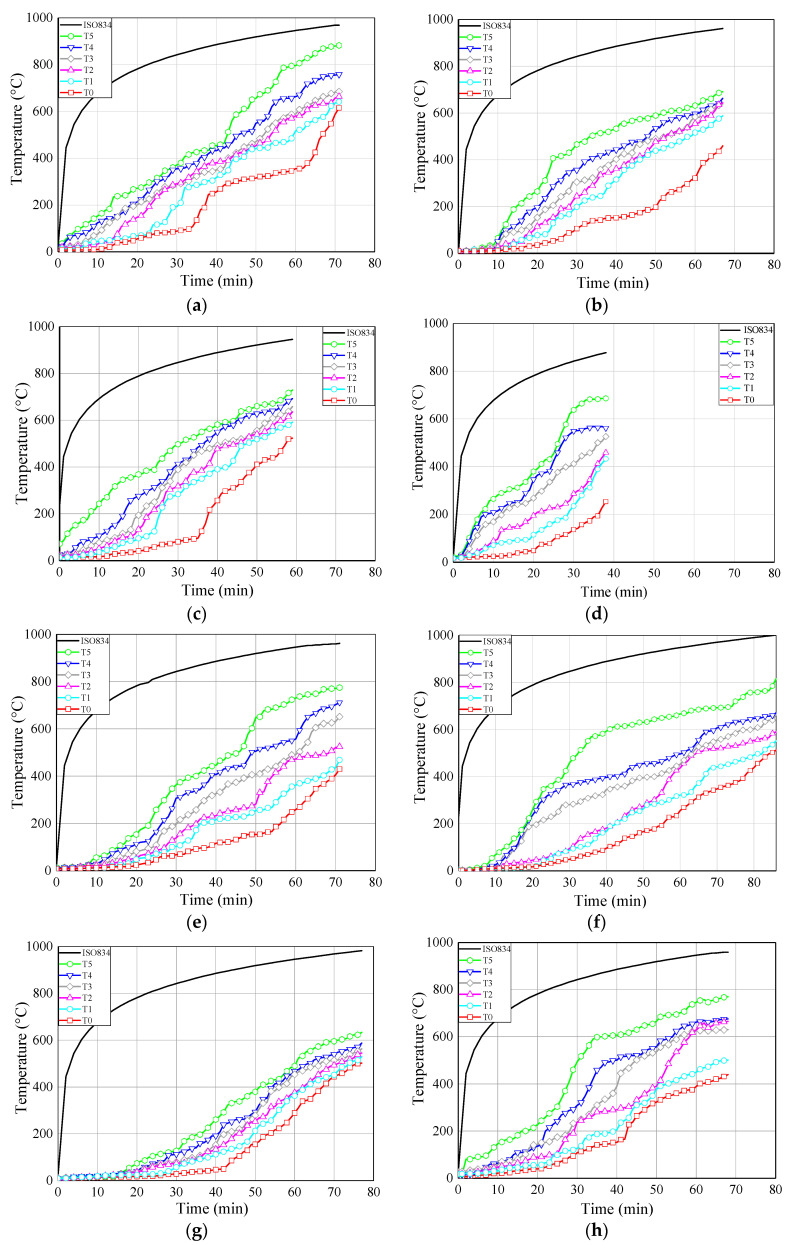
Temperature-time curves. (**a**) Z160-2; (**b**) Z160-3; (**c**) Z160-5; (**d**) S160-3; (**e**) Z200-3; (**f**) Z240-3; (**g**) ZF160-3; (**h**) ZTF160-3.

Figure 10 shows the temperature-time curves of some typical specimens for each group. The temperatures at the adhesive-rod interfaces were measured by thermocouples numbered T1, T2, T3, T4, and T5 (see Figure 5). The temperature at the centre of the timber block was measured by a thermocouple denoted T0. As shown in Figure 10, all of the temperature-time curves show the same trend.

With the increase of the distance from the loading end, the measured temperature decreased gradually; this is mainly owing to the heat transfer of the glued-in rod. Therefore, the fire protection and thermal insulation of exposed metal components, such as exposed rods in glued-in rod timber joints, is extremely significant in practical engineering, due to the relatively large thermal conductivity of metal components National Design Specifications (NDS) for wood construction (NDS-2018) [42] also states that the timber connection shall be protected from fire exposure for the required fire resistant time. Moreover, compared with bolted and dowel connections, the glued-in rod joints showed the least exposed metal component, and even can be sealed inside the preset notches by non-combustible plugging material [7], which showed advantage in the fireproof design.

It can be seen from Figure 10a–c that the stress level plays an important role in the failure time of the joint specimens. Therefore, the stress level should be kept within reasonable limits for the application of glued-in rod joints in fire protection design. By comparing Figure 10b,d, the joints employed modified epoxy resin (S160-3) showed pronounced improvement for the fire resistance that the joints using commercial epoxy resin (Z160-3), which denotes the proposed innovative adhesive effectively improve the fire resistance of epoxy adhesive. Therefore, it is of great significance to promote the engineering application of glued-in rod joints thanks to the improvement of fire resistance of modified epoxy adhesive. Moreover, with the increase of the sectional sizes and the introduction of the fireproof coating materials.

## 7. Discussion

### 7.1. Effects of Stress Level

**Figure 11:**
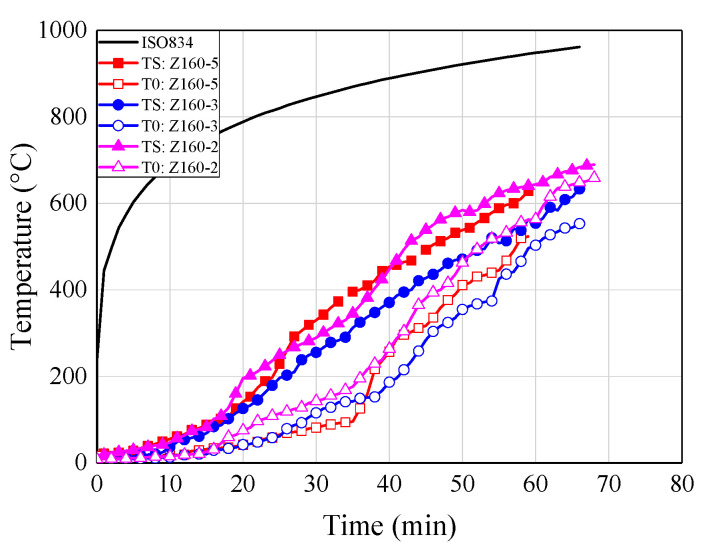
Effect of stress level.

**Figure 12:**
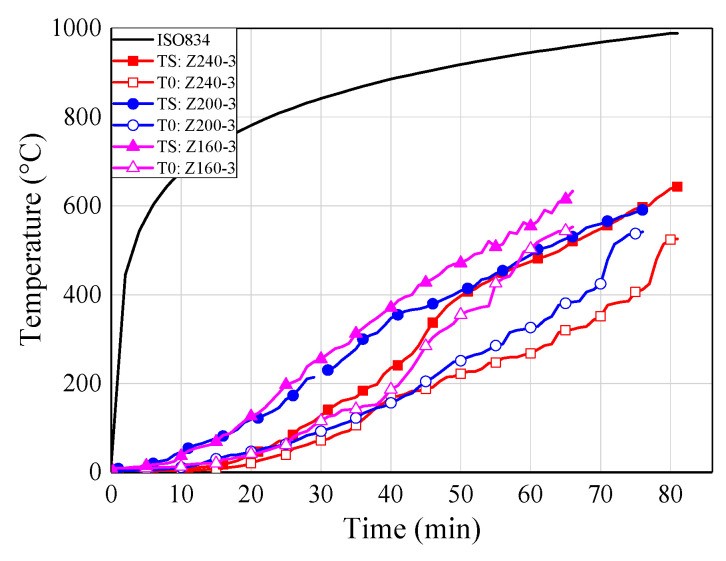
Effect of edge distance.

**Figure 13:**
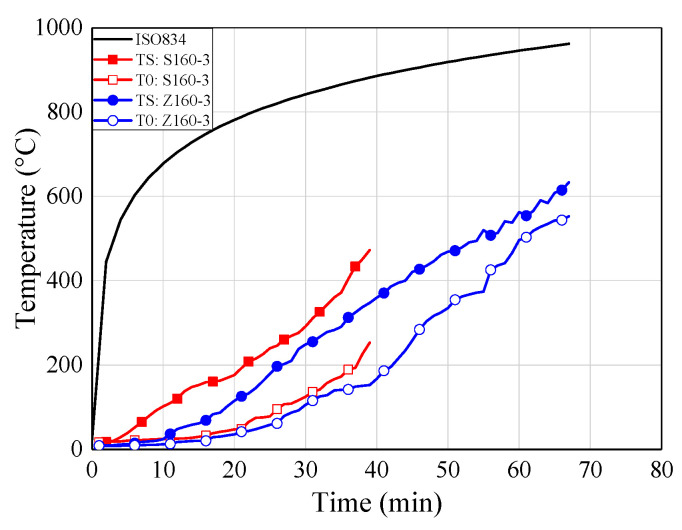
Comparison of different adhesives.

**Figure 14:**
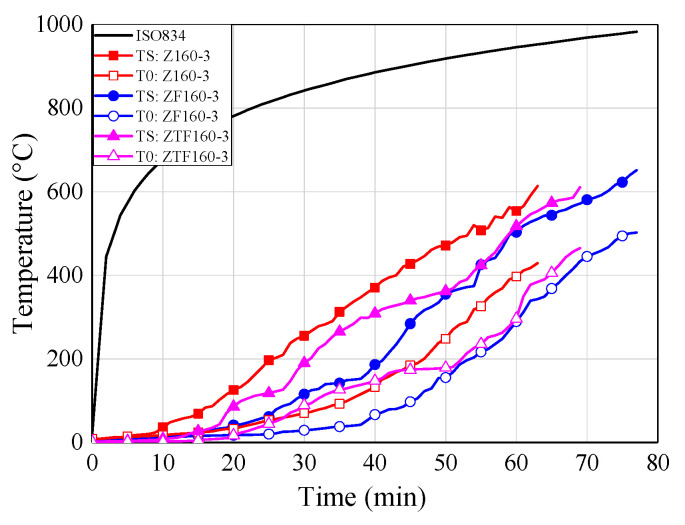
Comparison of different fireproof measures.

Figure 11 shows the effects of the stress level on the failure time of the glued-in rod joints in the fire tests. The temperature value of measuring point T0 refers to the average value of the three duplications. The values of TS were calculated by the 15 measuring points, i.e., T1~T5 for all of the three duplications. Same calculation method was also applying to Figure 12, Figure 13 and Figure 14. When the stress levels were 20%, 30%, and 50%, the corresponding average values for failure time were 70 min, 64 min, and 57 min, respectively. With the increase of the loading level, the fire resistance of glued-in rod joints declined obviously, denoting the decline of the heat resistant of the modified epoxy adhesive. Therefore, the stress level cannot be ignored in the fire protection design.

### 7.2. Effects of Edge Distance

The edge distance is changed by designing different sectional sizes, as shown in Table 5. For sectional sizes of 160 mm × 160 mm, 200 mm × 200 mm, and 240 mm × 240 mm, the corresponding edge distances were 80 mm, 100 mm, and 120 mm, respectively. Figure 12 shows a comparison of failure times among specimens with different edge distances. At the stress level of 30%, the average failure times of the above groups were 63 min, 78 min, and 82 min, respectively.

### 7.3. Comparison between the Different Adhesive

Figure 13 illustrates that the average refractory time for specimens with the modified epoxy adhesive was 62.3 min, whereas the average refractory time for those with the commercial epoxy resin was 37.3 min. Thus, the fire resistance period of the specimens using the modified epoxy resin increased from 37.3 min to 62.3 min as compared with the commercially available epoxy adhesive. In addition, the heating rate of the specimens using the modified epoxy resin was relatively lower than that of the specimens using commercially available epoxy adhesive.

### 7.4. Effects of Fireproof Measures

The temperature curves for different fireproof coating conditions are illustrated in Figure 14. The white coating showed a better fire-retardant improvement than the colourless fireproof coating. The white coating improves the fire resistance period for heat resistant modified epoxy adhesive by approximately 13 min, while the colourless coating by about 5 min. As shown in Table 6, for the specimen with commercial epoxy resin, the SF160-3 specimens showed a 20-min fire resistance improvement than S160-3 specimens due to the application of white coating. It can be concluded that the fire-retardant coating has a relatively slight effect on the modified epoxy adhesive compared with the commercial epoxy resin, thanks to the improvement in heat resistant of the modified epoxy adhesive.

## 8. Conclusions

In this work, an innovative modified fireproof epoxy resin adhesive was proposed, based on introducing some inorganic additives. Through tests both at room temperature and in fire, the effects on the fire resistance of joints from several influential factors were analysed in detail. The main conclusions of this study are summarised as follows:

- As far as mechanical behaviours are concerned, there is little difference between a glued-in rod timber joint with the heat-resistant modified epoxy resin and that with the commercial epoxy resin at room temperature.
- The fire resistance period of the specimens with the modified epoxy resin can be improved by 73.0% when compared with the specimens with the commercial epoxy resin.
- The fire resistance time declined to 57 min from 70 min, while the loading level in fire tests increased to 50% from 20%. The loading levels sustained by the stress component should be emphasized.
- When the specimens were protected by a white coating, the fire resistance period increased by 54.1% and 20.3% for the traditional and modified epoxy resin cases, respectively.
- As the joint edge distance changed from 5d to 7.5d, the failure time increased from 64 min to 83 min, an improvement of 29.7%.
- Under the same load level and sectional size, the specimens in group ZF160-3, which adopted the modified epoxy adhesive and white fire coating, showed the best fire resistance performance.

In conclusion, the modified epoxy adhesive showed the significant improvement in fire resistance compared with the ordinary commercial epoxy resin, without any decline in mechanical performance at room temperatures. Therefore, the modified epoxy adhesive proposed in this article exhibits superior application prospect in glued-in rod timber joints thanks to the advantage of heat resistant.

Further investigations will focus on the theoretical analysis about determining the temperature tendency of adhesive based on the charring rate and thermal conductivity of timber, as well as the stiffness reduction factor of glued-in rod joints with different adhesives in fires. Thus, the relationship among fire time, adhesive temperature, and joint stiffness can be established to guide the fire design of glued-in rods joints of timber structures.

## Figures and Tables

**Table 1:** Mechanical properties of glulam timber specimens (MPa).

Wood Designation (Parallel to Grain)	Values
Compressive strength	39.3
Tensile strength	28.6
Shear strength	9.2
Modulus of elasticity	1,1900

**Table 2:** Mechanical properties of threaded rod.

Threaded Rod	Values
Rod diameter (mm)	16
Tensile strength (MPa)	695
Yield strength (MPa)	842
Tensile modulus of elasticity (MPa)	2.03 × 10^5^

**Table 3:** Mechanical properties of the epoxy resin (MPa).

Mechanical Property	Values
Adhesion strength	11.2
Splitting tensile strength	13.6
Tensile shear strength	17.0

**Table 4:** Material ratios of modified epoxy adhesive.

Component A: Epoxy Resin Base Material		Component B: Inorganic Additive	
Ingredient	Portion	Ingredient	Portion
Epoxy E51	100	Modified amine hardeners JH5-55	50
Water-borne epoxy resin	50	Cement	80
Dibutyl phthalate (DBP) flexibilizer	10	Quartz sand	50
KH550 coupling agent	5	Silica powder	100
-	-	Carbon fibre powders	20

**Table 5:** Geometric parameters of specimens (mm).

Specimen Code	Sectional Sizes	Stress Level	Fireproof Coating	Adhesive Type
Z160-2	160 × 160	20%	No coating	Modified epoxy adhesive
Z160-3	160 × 160	30%	No coating	Modified epoxy adhesive
Z160-5	160 × 160	50%	No coating	Modified epoxy adhesive
ZF160-3	160 × 160	30%	White coating	Modified epoxy adhesive
ZTF160-3	160 × 160	30%	Colourless coating	Modified epoxy adhesive
Z200-3	200 × 200	30%	No coating	Modified epoxy adhesive
Z240-3	240 × 240	30%	No coating	Modified epoxy adhesive
S160-3	160 × 160	30%	No coating	Commercial epoxy resin
SF160-3	160 × 160	30%	White coating	Commercial epoxy resin

**Table 6:** The failure time of all specimens.

Groups	Failure Time (min)/Failure Modes			Average Failure Time (min)
	Specimen 1	Specimen 2	Specimen 3	
Z160-2	71/A	67/A	72/A	70
Z160-3	67/A	61/A	63/A	64
Z160-5	57/A	54/A	60/A	57
ZF160-3	74/A	80/A	77/A	77
ZTF160-3	68/A	66/A	71/A	68
Z200-3	72/A	76/A	16/B	74
Z240-3	85/A	82/A	81/A	83
S160-3	38/A	34/A	40/A	37
SF160-3	61/A	12/B	53/A	57

Note: Failure mode A denotes the pull-out of the rod, whereas failure mode B denotes the fracture of the rod owing to the heat of the fire.

## References

[1] Liu W., Yang H. (2019). Research progress of modern wood structures. J. Build. Struct..

[2] Schweigler M., Bader T.K., Hochreiner G., Lemaître R. (2018). Parameterization equations for the nonlinear connection slip applied to the anisotropic embedment behavior of wood. Compos. Part B Eng..

[3] Loss C., Hossain A., Tannert T. (2018). Simple cross-laminated timber shear connections with spatially arranged screws. Eng. Struct..

[4] Ling Z., Liu W., Lam F., Yang H., Lu W. (2015). Bond behavior between softwood glulam and epoxy bonded-in threaded steel rod. J. Mater. Civil Eng..

[5] Steiger R., Serrano E., Stepinac M., Rajčić V., O’Neill C., McPolin D., Widmann R. (2015). Strengthening of timber structures with glued-in rods. Constr. Build. Mater..

[6] Schober K.U., Tannert T. (2016). Hybrid connections for timber structures. Eur. J. Wood Wood Prod..

[7] Nouri F., Valipour H. (2019). Semi-rigid partial-strength steel-timber composite (STC) connections with mechanically anchored steel rods. J. Constr. Steel Res..

[8] Yang H., Tang L., Chen Y., Zhang Y., Tao H., Liu W., Hu J. (2020). Experimental and theoretical behaviour of composite joints between timber-concrete beam and column. J. Build. Struct..

[9] Broughton J.G., Hutchinson A.R. (2001). Pull-out behaviour of steel rods bonded into timber. Mater. Struct..

[10] Chans D.O., Cimadevila J.E., Gutiérrez E.M. (2009). Influence of the geometric and material characteristics on the strength of glued joints made in chestnut timber. Mater. Design..

[11] Yeboah D., Taylor S., McPolin D., Gilfillan R., Gilbert S. (2011). Behaviour of joints with bonded-in steel bars loaded parallel to the grain of timber elements. Constr. Build. Mater..

[12] Widmann R., Steiger R., Gehri E. (2007). Pull-out strength of axially loaded steel rods bonded in glulam perpendicular to the grain. Mater. Struct..

[13] Azinović B., Serrano E., Kramar M., Pazlar T. (2018). Experimental investigation of the axial strength of glued-in rods in cross laminated timber. Mater. Struct..

[14] Vallée T., Adam M. (2016). Inductively cured glued-in rods in timber using Curie particles. Int. J. Adhes. Adhes..

[15] Ling Z., Yang H., Liu W., Zhou D., Wang L. (2014). Pull-out strength and bond behaviour of axially loaded rebar glued-in glulam. Constr. Build. Mater..

[16] Ling Z., Xiang Z., Liu W., Yang H., Tang J. (2019). Load-slip behaviour of glue laminated timber connections with glued-in steel rod parallel to grain. Constr. Build. Mater..

[17] Fragiacomo M., Batchelar M. (2011). Timber frame moment joints with glued-in steel rods. I: Design. J. Struct. Eng..

[18] Fragiacomo M., Batchelar M. (2011). Timber frame moment joints with glued-in steel rods. II: Experimental investigation of long-term performance. J. Struct. Eng..

[19] Xu B., Guo J., Bouchaïr A. (2020). Effects of glue-line thickness and manufacturing defects on the pull-out behavior of glued-in rods. Int. J. Adhes. Adhes..

[20] Grunwald C., Vallée T., Fecht S., Bletz-Mühldorfer O., Diehl F., Bathon L., Myslicki S., Scholz R., Walther F. (2019). Rods glued in engineered hardwood products part I: Experimental results under quasi-static loading. Int. J. Adhes. Adhes..

[21] Grunwald C., Vallée T., Fecht S., Bletz-Mühldorfer O., Diehl F., Bathon L., Walther F., Scholz R., Myslicki S. (2019). Rods glued in engineered hardwood products part II: Numerical modelling and capacity prediction. Int. J. Adhes. Adhes..

[22] He Z., Xiao Y. (2020). Experimental study on axial pull-out behavior of steel rebars glued-in glubam. J. Mater. Civ. Eng..

[23] Maraveas C., Miamis K., Matthaiou C.E. (2015). Performance of timber connections exposed to fire: A review. Fire Technol..

[24] Jessop D., Abu A., Wade C., Spearpoint M., Gerlich H. (2019). Performance of a light timber—framed compartment in natural fire subjected to lateral load. Fire Mater..

[25] Peng L., Hadjisophocleous G., Mehaffey J., Mohammad M. (2012). Fire performance of timber connections, part 1: Fire resistance tests on bolted wood-steel-wood and steel-wood-steel connections. J. Struct. Fire Eng..

[26] Moss P.J., Buchanan A.H., Fragiacomo M., Lau P.H., Chuo T. (2009). Fire performance of bolted connections in laminated veneer lumber. Fire Mater..

[27] Akotuah A.O., Ali S.G., Erochko J., Zhang X., Hadjisophocleous G.V. Study of the fire performance of hybrid steel-timber connections with full-scale tests and finite element modelling. Proceedings of the International Conference on Applications of Structural Fire Engineering (ASFE 2015).

[28] Audebert M., Dhima D., Taazount M., Bouchaïr A. (2012). Behavior of dowelled and bolted steel-to-timber connections exposed to fire. Eng. Struct..

[29] Audebert M., Dhima D., Taazount M., Bouchaïr A. (2013). Thermo-mechanical behaviour of timber-to-timber connections exposed to fire. Fire Safety J..

[30] Audebert M., Dhima D., Taazount M., Bouchaïr A. (2014). Experimental and numerical analysis of timber connections in tension perpendicular to grain in fire. Fire Safety J..

[31] Erchinger C., Frangi A., Fontana M. (2010). Fire design of steel-to-timber dowelled connections. Eng. Struct..

[32] Liu J., Yue. K., Xu. L., Wu J., Chen Z., Wang L., Liu W., Lu W. (2020). Bonding performance of melamine-urea–formaldehyde and phenol-resorcinol–formaldehyde adhesive glulams at elevated temperatures. Int. J. Adhes. Adhes..

[33] Östman B.A.-L. (2017). Fire performance of wood products and timber structures. Int. Wood Prod. J..

[34] ANSI/AWC Manual for Wood Engineered Wood Construction.

[35] Ni C., Popovski M. (2015). Mid-Rise Wood-Frame Construction Handbook.

[36] Harada T., Kamikawa D., Miyatake A., Shindo K., Hattori N., Ando K., Miyabayashi M. (2019). Two-hour fireproof performance of cross laminated timber (CLT) covered with fire-retardant impregnated wood. Mokuzai Gakkaishi.

[37] Yue K., Wu J., Xu L., Tang Z., Chen Z., Liu W., Wang L. (2020). Use impregnation and densification to improve mechanical properties and combustion performance of Chinese fir. Constr. Build. Mater..

[38] Karacabeyli E., Lum C. (2014). Technical Guide for the Design and Construction of Tall Wood Buildings in Canada.

[39] Popescu C.M., Pfriem A. (2020). Treatments and modification to improve the reaction to fire of wood and wood based products—An overview. Fire Mater..

[40] Frangi A., Knobloch M., Fontana M. (2010). Fire design of timber-concrete composite slabs with screwed connections. J. Struct. Eng..

[41] CEN (European Committee for Standardization) (2004). Eurocode 5: Design of Timber Structures—Part 1-1: General—Common Rules and Rules for Buildings, EN 1995-1-1.

[42] ANSI/AWC (2018). National Design Specifications (NDS) for Wood Construction.

[43] Lartigau J., Coureau J., Morel S., Galimard P., Maurin E. Effect of temperature on load bearing capacity of glued-in rods. Proceedings of the World Conference on Timber Engineering 2012 (WCTE 2012).

[44] Harris S. (2004). Fire Resistance of Epoxy-grouted steel rod connections in laminated veneer lumber (LVL). Master’s Thesis.

[45] Park J.S., Buchanan A.H., Lee J.J. (2006). Fire performance of laminated veneer lumber (LVL) with glued-in steel rod connections. J. Fire Sci..

[46] ASTM (American Society for Testing and Materials) (2002). Standard Test Methods of Static Tests of Lumber in Structural Sizes ASTM D 198-02.

[47] CEN (European Committee for Standardization) (2003). Timber Structures—Structural Timber and Glued Laminated Timber—Determination of Same Physical and Mechanical Properties EN 408: 2003.

[48] Shi B., Liu W., Yang H., Ling X. (2020). Long-term performance of timber-concrete composite systems with notch-screw connections. Eng. Struct..

[49] ASTM (American Society for Testing and Materials) (2013). Standard Test Method for Strength Properties of Adhesive Bond in Shear by Compression Loading D 905-08(2013).

[50] CEN (European Committee for Standardization) (2002). Moisture Content of a Piece of Sawn Timber—Part 1: Determination by Oven Dry Method, EN 13183-1:2002.

[51] (1994). GB/T 15389: Threaded Rods. General Administration of Quality Supervision.

[52] (2010). GB/T 228: Chinese Standard for Tensile Tests of Metallic Materials. General Administration of Quality Supervision.

[53] (2018). GB 12441: Finishing Fire Retardant Coating. General Administration of Quality Supervision.

[54] (1999). ISO 834-1: Fire-Resistance Tests—Elements of Building Construction—Part 1: General Requirements.

